# Management measures for non-medical staff on psychiatric hospital wards during the COVID-19 pandemic

**DOI:** 10.1192/bjb.2020.65

**Published:** 2020-08

**Authors:** Lei Yang, Dongmei Wu

**Affiliations:** Assistant Professor, Chengdu Medical College, China, email: tastylei@126.com; Associate Professor, The Clinical Hospital of Chengdu Brain Science Institute, China, email: wudongmei_2001@163.com

A notice issued by the National Health Commission in China on 18 March 2020 highlighted the need for psychological counselling for those affected by COVID-19, vulnerable groups, health workers and those fighting the virus on the frontline.^1^ Data shows that 323 patients with severe mental disorders had been diagnosed with COVID-19 across the country as of 19 February 2020.^2^ The development of an epidemic poses great challenges for psychiatric hospitals.

There are visitors and non-medical staff who work in hospitals, such as cleaners, who have no medical experience or knowledge about prevention of infectious diseases. In order to ensure the safety of in-patients and staff, it is necessary to formulate management measures for non-medical staff.

First, there needs to be strict implementation of a system for non-medical staff to sign in and out of the ward. The following details should be entered into a table: name, date of going out, time of going out, reason for going out, estimated return time, actual return time, temperature at return, exact location of outing, etc. They can leave the ward only after a nurse has signed it.

Second, it is essential to follow procedures formulated by the wards and service providers. Wards should undertake temperature monitoring of non-medical staff at least twice a day. Wards should be responsible for monitoring the non-medical staff's work, including the wearing of protective clothing and ensuring that proper hand washing is carried out. Cleaners are required to clean and disinfect tableware and dining tables after meals. Staff need to strictly implement management regulations when transporting patients’ specimens. Unless essential, wards and service providers should not replace permanent staff with temporary or agency staff. There must be epidemiological screening of new recruits to ensure that these personnel do not have a history of epidemiological exposure, fever or any respiratory symptoms.

Third, wards need to provide training and guidance. The ward's head nurse needs to consider the different educational levels of non-medical staff when developing and undertaking training regarding epidemic prevention knowledge and skills to ensure all training is appropriate. The training needs to focus on disinfection, isolation, hand hygiene and wearing protective clothing. The responsible nurse is in charge of daily guidance for non-medical staff. When problems are identified with epidemic prevention they should correct issues without delay.

Fourth, wards and service providers need to work together on management supervision. The ward's head nurse needs to evaluate the impact of training on non-medical staff daily. Service provider managers need to check the results of training and keep corresponding records. The head nurse of the department needs to supervise the work of non-medical staff in wards under their jurisdiction. Then, they need to check the preventive knowledge and skills of the non-medical staff members and report the results to the director of nursing. The chief manager needs to undertake special inspections to assess the mastery of epidemic preventive knowledge, skills and the quality of work in the hospital on a weekly basis.

Finally, it is important to keep an eye on the psychological status of non-medical staff on wards. On account of the long-term exposure to a dangerous environment, coupled with patients’ uncooperative attitudes, loud yelling or violent behaviour, their visitors and caregivers are prone to psychological difficulties.^3^ As cleaners frequently enter or leave wards, the exposure risk increases. If there are any signs of anxiety or panic, non-medical staff should receive psychological interventions to help them properly handle negative feelings.

Recently, the number of cases of COVID-19 outside of China has become very serious. This letter aims to formulate epidemic prevention and control measures for non-medical workers in psychiatric hospitals. It is hoped that these measures will be of value for both domestic and foreign epidemic prevention efforts.

## References

[1] National Health Commission of the People's Republic of China. Mental Health Highlighted as China sees Progress in Epidemic Control. China NHC, 2020. Available from: http://en.nhc.gov.cn/2020-03/20/c_78022.htm.

[2] National Health Commission of the People's Republic of China. Psychiatric Hospitals Should Better Care for Mental Patients during Novel Coronavirus Outbreak. China NHC, 2020. Available from: http://en.nhc.gov.cn/2020-02/19/c_76701.htm.

[3] Hilton NZ, Ham E, Dretzkat A. Psychiatric hospital workers' exposure to disturbing patient behavior and its relation to post-traumatic stress disorder symptoms. Can J Nurs Res 2017; 49: 118–26. 28841065 10.1177/0844562117719202

